# Active Breaks Enhance Complex Processing Speed, Math Performance, and Physical Activity in Primary School Children: A Randomized Controlled Trial

**DOI:** 10.3390/jfmk10040376

**Published:** 2025-09-29

**Authors:** Giovanni Fiorilli, Gloria Di Claudio, Domenico Di Fonza, Francesca Baralla, Giovanna Aquino, Giulia Di Martino, Carlo Della Valle, Marco Centorbi, Giuseppe Calcagno, Andrea Buonsenso, Alessandra di Cagno

**Affiliations:** 1 Department of Medicine and Health Sciences, University of Molise, 86100 Campobasso, Italy; fiorilli@unimol.it (G.F.); gloria.diclaudio@univr.it (G.D.C.); difonzad@gmail.com (D.D.F.); francesca.baralla@unimol.it (F.B.); giovanna.aquino@unimol.it (G.A.); giulia.dimartino21@gmail.com (G.D.M.); carlo.dellavalle@univr.it (C.D.V.); giuseppe.calcagno@unimol.it (G.C.); 2 Department of Neurosciences, Biomedicine and Movement, University of Verona, 37129 Verona, Italy; 3 Department of Theoretical and Applied Sciences, eCampus University, 22060 Como, Italy; andrea.buonsenso@uniecampus.it; 4 Department of Movement, Human and Health Sciences, University of Rome “Foro Italico”, 00135 Rome, Italy; alessandra.dicagno@uniroma4.it; 5 Department of Human Sciences, Guglielmo Marconi University, 00193 Rome, Italy

**Keywords:** executive functions, school-based intervention, academic achievement, active breaks

## Abstract

**Objectives**: This study aimed to evaluate the effects of a 12-week Active Breaks (ABs) program on physical, cognitive, and academic outcomes in primary school children. **Methods**: Eighty primary school students (age: 7.52 ± 0.50) (BMI: 18.35 ± 3.07) were recruited and randomly assigned to three experimental groups—involving creativity-based (CRE) (age: 7.97 ± 0.18 years) (BMI: 20.01 ± 3.59), fitness-based (FIT) (age: 7.93 ± 0.26 years) (BMI: 16.74 ± 1.76), and combined (COM) (age: 7.97 ± 0.18 years) (BMI: 19.38 ± 4.24) ABs—and a control group (CON) (age: 7.42 ± 0.49 years) (BMI: 18.31 ± 2.64). The intervention consisted of two daily sessions (10 min each) three times per week over a 12-week period. Numerical skills, calculation abilities, and arithmetic problem-solving performance were evaluated using the “Test for the Assessment of Calculation and Problem-Solving Skills” (AC-MT 6-11). Attention and concentration performance were assessed using the Reynolds Interference Task (RIT). Motor skill performance was assessed using the MOTORFIT tests. **Results**: The FIT and CRE groups showed higher improvement in physical performances (*p* < 0.05). Regarding cognitive outcomes, the COM group outperformed the CON group in the Total Correct Index (*p* = 0.032). Regarding mathematical performance, all EGs achieved higher results than the CON group (*p* < 0.042), with the COM group achieving the highest scores in operations, problem-solving, and total scores (*p* < 0.032). **Conclusions**: Incorporating structured physical activity through ABs during curricular hours is an effective strategy to enhance physical, cognitive, and academic performance in primary school children. A combined approach appears to be especially beneficial, supporting both physical and cognitive development simultaneously.

## 1. Introduction

Although Physical Education (PE) is recognized as crucial, particularly in primary schools, the time allocated to PE is often insufficient and varies considerably across contexts, with classes frequently delivered by non-specialist personnel. This insufficient exposure fails to meet the daily PA required to maintain children’s health [1]. Several studies have indicated that moderate-to-vigorous physical activity (MVPA) in school-aged children is closely related to physical, mental, and social health benefits [2]. Across European nations, there is widespread low adherence to physical activity (PA), with many countries in the European Union experiencing a consistent increase in the number of inactive children, indicating noncompliance with the guidelines. This trend is particularly notable in Italy, where only 9.5% of boys and 2.6% of girls meet the daily requirements [3,4].

The introduction of PE in schools faces significant barriers, often due to curriculum demands and academic accountability pressures [5]. Nevertheless, considering that children spend a considerable amount of time within the educational setting, the classroom constitutes an optimal context for the promotion of PA among school-aged populations. Therefore, Active Breaks (ABs) are currently receiving increased attention in educational settings [6]. ABs involve brief intervals, typically ranging from 5 to 15 min, of PA that is conducted within the classroom setting [7], integrated into the daily routine [6,8], and delivered by classroom teachers either between lessons or integrated into the lessons themselves [9]. ABs offer several advantages, such as not requiring special spaces or equipment, and the flexibility to choose when to integrate Abs, creating a more engaging learning environment. A recent study demonstrated the effectiveness of ABs as a strategy to improve children’s attention and behavior in primary school settings. The findings suggest that incorporating structured physical activity breaks not only enhances cognitive functioning but also supports more sustainable educational practices [10].

Several studies have examined both the acute [11] and chronic [12] effects of ABs on academic performance and motor skills, highlighting improvements in working memory, attention, and processing speed and the positive impact on students’ cognitive, metacognitive, and academic outcomes [13]. These brain-related improvements in learning outcomes are achieved using methodologies that emphasize active student engagement [14]. Among cognitive outcomes, attention plays a crucial role in supporting different dimensions of learning and the consolidation of memory [15]. Engaging in short bursts of physical activity promotes the release of neurotransmitters, enhancing physiological arousal and attention levels, thereby positively influencing cognitive performance. Based on this evidence, the present study aimed to evaluate the effects of a 12-week AB program on physical, cognitive, and academic outcomes in primary school children.

Specifically, this study represents the first investigation into the effects of three distinct AB protocols: one emphasizing creativity-based exercises, one centered on fitness-oriented activities, and one integrating both creative and fitness components. It was hypothesized that the combined group would achieve superior outcomes relative to the other experimental and control groups, likely attributable to the synergistic interaction of cognitive and physical stimulation.

## 2. Materials and Methods

### 2.1. Study Design

A mixed experimental design was used to compare three experimental conditions and one control condition, measured at two time points (baseline and post-test), to assess the physical, attentional, and mathematical performance of primary school children. Baseline assessment data were collected during the week prior to the start of the intervention. At the end of the 12-week intervention, the same procedures were used to collect post-test outcomes through tests evaluating numerical skills, calculation abilities, and arithmetic problem-solving, as well as tests assessing attention and concentration, followed by a battery of motor skill evaluations. All interventions were implemented during regular class time by qualified researchers, with the active involvement of teachers. Prior to the intervention, teachers participated in several training sessions designed to foster a shared understanding of the intervention’s strategies and its significance for health promotion and cognitive enhancement. These sessions also sought to increase adherence to the project among both teachers and students. For this purpose, teachers were provided with structured programs and evidence-based methodologies.

#### Participants

Eighty students were recruited for the study from primary schools in Campobasso (Italy). After the baseline assessment, the classes were randomly assigned to three experimental groups (EGs) distinguished by the type of protocol performed and a control group (CON) that did not participate in any AB intervention: creativity-based AB (CRE; n = 20), fitness-based AB (FIT; n = 20), combined AB (COM; n = 20), and control group (CON; n = 20).The ABs were performed twice on the same school day for 10 min per AB session, three times per week for 12 weeks. Sample characteristics are shown in Table 1.

The only exclusion criterion was the presence of injuries that could prevent physical participation.

During the experimental procedures, participants who missed at least 24 AB sessions (for a total of 72 AB sessions) were excluded from the statistical analysis; moreover, no participants dropped out. The school principal, teachers, and parents of the participating classes received an information statement and were informed of the study’s purpose. All students participated in the program as part of a whole-class intervention. The parents of the children provided written informed consent.

This study was designed and conducted in accordance with the Declaration of Helsinki and was approved by the bioethical local committee of University of Rome “Foro Italico” (University Committee for Research (CAR-IRB), (Code: 61/2020). This study was registered at www.clinicaltrials.gov (NCT06684808).

### 2.2. Procedures

Motor skill performance was evaluated using the MOTORFIT tests [16]. Numerical skills, calculation abilities, and arithmetic problem-solving were assessed with the “Test for the Assessment of Calculation and Problem-Solving Skills” (AC-MT 6-11) [17]. Attention and concentration were measured using the Reynolds Interference Task (RIT) [18]. Exercise intensity and perceived exertion were monitored using the Rating of Perceived Exertion (RPE) scale, whose scores range from 1 to 10 [19].

Before administering the tests, each child’s anthropometric data were assessed. Height was measured via a Stadiometer (SECA 213) in a standing position with shoes removed, shoulders relaxed, facing forward with head and back facing the wall. Weight was measured with minimal clothing on using a Tanita TBF-310GS Total Body Composition Analyzer. BMI was calculated as weight (kg) divided by height squared (m^2^).

All measurements were conducted under standardized conditions in a designated free room within the school to minimize systematic errors and enhance the reliability of the assessments.

**Figure 1:**
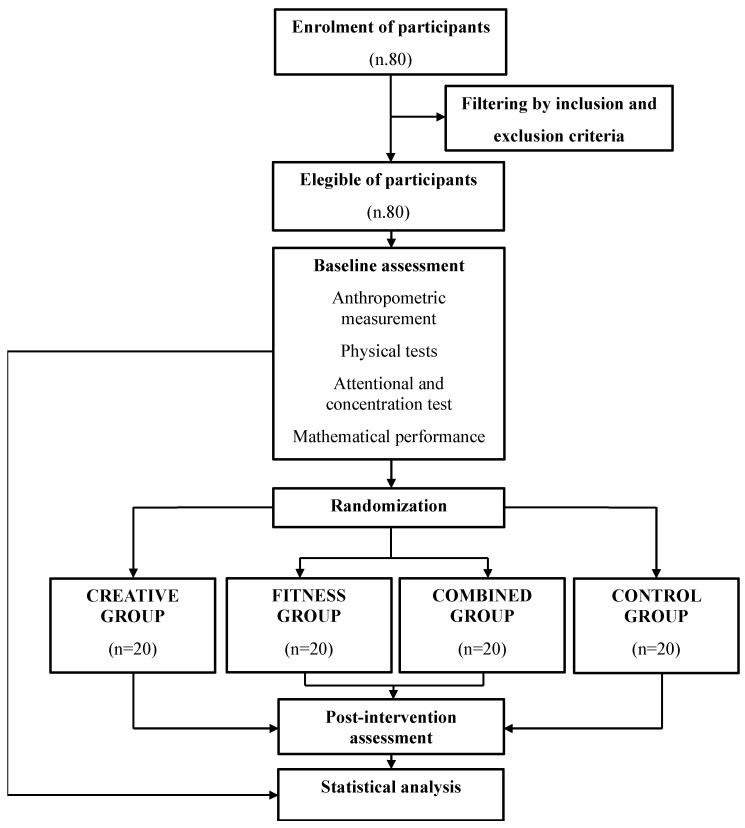
CONSORT flow diagram of the study.

The CONSORT flow diagram of the study is shown in Figure 1.

#### 2.2.1. Motorfit

Motor skills were evaluated using the Motorfit Test Battery, a standardized assessment consisting of a series of targeted exercises designed to measure distinct components of motor performance.

– Forward hopping on one foot (FHF-1): assesses balance, unilateral coordination, and lower-limb strength.
– Lateral gallop (LG): measures agility, lateral coordination, and rhythm.
– Alternating forward hopping on one foot (FHF-2): evaluates dynamic balance, inter-limb coordination, and rhythmic control.
– Throwing a ball with one hand (TBH): assesses upper-limb coordination, strength, and object manipulation skills.
– Catching a thrown ball (CB): evaluates hand–eye coordination and reaction time.
– Hitting a ball with a tennis racket (HB): measures precision, coordination, and bimanual control.
– 10 × 5 m shuttle run (VEL): assesses speed, agility, and ability to change direction.
– Long jump from a standing start (LJ): evaluates explosive lower-body strength and bilateral coordination.

Each test was given a score between 0 and 4 based on the student’s performance. The raw data are used for statistical analysis. This test battery evaluates different fundamental physical abilities essential for psychomotor development, including coordination, strength, endurance, and other key motor skills.

#### 2.2.2. Test for the Assessment of Calculation and Problem-Solving Skills (AC-MT 6-11)

The AC-MT 6-11 is a standardized assessment battery designed to evaluate numerical skills, calculation abilities, and arithmetic problem-solving in children aged 6 to 11 years within educational settings.

The battery is designed to assess core cognitive domains fundamental to mathematical learning. It consists of multiple subtests, each targeting a specific domain.

– Operations (OPS): assesses fluency and accuracy in performing fundamental arithmetic operations, which serve as the basis for more-advanced mathematical competencies.
– Numerical Judgment (NJ): evaluates intuitive numerical reasoning, essential for the development of mental number representation and estimation strategies.
– Tens and Units Task (TU): measures understanding of place value, a critical construct for arithmetic proficiency and multidigit number manipulation.
– Ordering Tasks (OTs): including both largest-to-smallest and smallest-to-largest sequences, assess visual working memory and serial ordering abilities for numerical information.
– Mathematical Problems (MP): examine higher-order cognitive processes by requiring integration of executive functions, working memory, and numerical reasoning, thereby reflecting applied mathematical problem-solving in real-world contexts.

The final score (TOTAL SCORE, TS) was obtained by summing the scores of the five subtests [17].

#### 2.2.3. Reynolds Interference Task (RIT)

The RIT was developed to assess cognitive processing speed more comprehensively than traditional tests. It is suitable for individuals aged 6 to 94 years and consists of two subtests:

– Object interference (OI = 60 s), which assesses semantic inhibition, perceptual–attentional control, verbal processing, and object recognition.
– Color interference (CI = 90 s), which measures inhibitory control, selective attention, and processing speed.

The scores obtained on these subtests contribute to the calculation of the Index Total Correct (ITC) index:

ITC = Correct responses on CI + Correct responses on OI

This index provides a global measure of cognitive efficiency under interference, integrating aspects of selective attention, processing speed, response inhibition, and cognitive control.

The OI subtest was administered with a time limit of 60 s, and the total number of correct responses within this window served as the primary raw score. Additionally, each subject completed the subtest without time restriction, and performance was evaluated based on the number of errors (E OI PO) and completion time (T OI PO).

The CI subtest was administered with a time limit of 90 s, with the total number of correct responses also serving as the primary raw score. As with OI, each subject completed the subtest without time restriction and performance was further assessed using error count (E CI PO) and completion time (T CI PO).

#### 2.2.4. Rate of Perceived Exertion (RPE)

RPE is a scale used to quantify the subjective perception of physical effort, providing a useful parameter for determining or adjusting the exercise intensity [19].

In this study, the RPE scale was introduced to children through a detailed explanation of the meaning of each effort level, ranging from 1 to 10, with images. At the end of each active break, the participants were asked to assign a value between 1 and 10 to indicate their perceived level of exertion.

### 2.3. Intervention

The PA intervention consisted of two AB sessions conducted on the same school day, each lasting 10 min, totaling 60 min per week for 12 weeks. The classes were randomly assigned to three AB protocols: CRE, FIT, and COM.

The CRE protocol focused on developing cognitive functions through the integration of creative movements and interactive games. The students were engaged in activities that stimulated their imagination, problem-solving skills, and critical thinking through exercises. The activities included role-playing, interpretive dance, and free movement associated with specific cognitive tasks. The goal was to combine cognitive learning with movement to make the educational process dynamic and engaging. The FIT protocol involved physical activities ranging from moderate to intense exercise, including aerobic activities (such as running in place and jumping) and strength and endurance exercises (such as push-ups and squats). The COM protocol is a combination of the first two approaches, involving the execution of a creative protocol followed by a fitness protocol. This protocol aimed to provide a balanced experience that stimulated students’ cognitive and physical abilities. The combination of the two types of breaks allowed alternating moments of high physical intensity with periods of lighter, more creative activities, promoting a holistic approach to school well-being. All protocols were accompanied by music. Perception of physical effort was monitored through the RPE scale, which was also used weekly to increase the intensity of the AB protocol. In the CON group, the children did not participate in any AB protocols, but they completed the regular physical activity directed by the teacher as part of the curriculum.

### 2.4. Statistical Analysis

Statistical analyses were performed using SPSS software version 23.0 (IBM, Chicago, IL, USA). Variables were tested for normal distribution using the Shapiro–Wilk test. The significance level for the statistical tests was set at 0.05. Descriptive statistics are reported as mean and standard deviation (SD). As the variables followed a normal distribution, analysis of variance for repeated measures (RM-ANOVA) was conducted to assess between-group (FIT vs. CRE vs. COM vs. CON) and within-group (pre-vs. post-intervention) differences. Additionally, a time*group interaction was calculated. Post-hoc comparisons were performed using the Bonferroni test. The independent variables were the scores obtained in the MotorFit test (FHF-1, FHF-2, LG, TBH, CB, HB, VEL, and LJ), cognitive test (ITC, E OI PO, T OI PO, E CI PO, T CI PO), and mathematical performance (OPS, NJ, TU, OT, MP, and TOTAL SCORE). In addition, the partial eta square (η2p) was calculated as an indicator of the effect size of the analysis. A partial eta-squared value between 0.01 and 0.06 indicates a small effect size, a value between 0.06 and 0.13 indicates a medium effect size, and a value equal to or higher than 0.14 indicates a large effect size [20].

The sample size was calculated using G*Power (version 3.1.9.7; written by Franz Faul, University of Kiel, Kiel, Germany). The following design specifications were considered: test family = F tests; statistical test = repeated measures of analysis of variance (ANOVA) between factors; α = 0.05; (1 − β) = 0.95; effect size f = 0.5; number of groups = 4; number of measurements = 2. The sample size estimation indicated that 56 participants were required, with a critical F-value of 2.782.

## 3. Results

### 3.1. Physical Performance Results

Significant differences in time were observed in all experimental groups compared to their pre-assessment measurements in the hopping tests (FHF-1 and FHF-2), while the CON group remained substantially unchanged. In HB test, the CRE and COM groups showed increased performance compared to their pre-assessment measurements.

Both the VEL and LJ tests showed statistically significant performance improvements in the FIT and COM groups when compared to their pre-assessment measurements (*p* < 0.001).

Significant between-group differences were found, with the FIT and CRE groups outperforming the CON group in FHF-1, FHF-2, TBH and HB (*p* < 0.05). Moreover, the CRE group outperformed the COM group in the LJ test (*p* = 0.044)

The results are shown in Table 2.

### 3.2. Cognitive Performance Results

All EGs showed significant improvements between pre- and post-assessments in the RIT test (ITC), as well as in the OI subtests (after 60 and 90 s), specifically in terms of error count (E OI PO) and completion time (T CI PO). The control group remained substantially unchanged.

A significant difference was found between the groups in ITC, where the COM group achieved a higher score than the CON group (*p* = 0.032).

The results are shown in Table 3.

### 3.3. Mathematical Performance Results

A statistically significant reduction in completion time was found in the OPS, MP, and TS measures, with all experimental groups showing enhanced performance compared to their pre-assessment measurements (*p* < 0.001).

Significant between-group differences were found in OPS, NJ, and MP, with the COM group scoring significantly higher than the CON group (*p* < 0.032). Regarding the total score, all experimental groups outperformed the CON group (*p* < 0.042)

The results are shown in Table 4.

## 4. Discussion

The study, which integrated one hour of weekly PA through six 10-min activity breaks, demonstrated significant improvements in physical efficiency and cognitive functions, including numerical calculation skills, arithmetic problem-solving, and cognitive processing speed. These effects are likely attributable to the ability of PA to stimulate and energize children. Such stimulation may help them maintain focus during school activities, which could induce a cognitive fatigue [21]. Scientific evidence confirms the validity of integrating AB protocols into the daily school curriculum and demonstrates positive effects on attention and mathematical performance—even following a single session of an AB program [11].

Motor assessment revealed a significant improvement across hopping, hitting, strength, and agility domains in EGs. In particular, the FIT and CRE groups showed superior performances compared to the CON group, which remained substantially stable throughout the entire experimental period. This finding confirmed that an increase in the weekly amount of PA is associated with greater improvements in children’s motor performance [22,23].

Our finding indicated that the COM group showed greater improvements in explosive strength areas, where structured protocols were more effective than the CRE protocol. Considering the distinct protocols implemented in the EGs (pre-/post-evaluation), the tests assessing strength and speed revealed more-pronounced improvements in the FIT and COM groups. In contrast, in the hitting test, which places greater emphasis on cognitive components, the CRE group demonstrated the most significant improvement. These results underscore the importance of incorporating mixed-intensity (high- and medium-intensity) PA into children’s daily routines to foster the development of complex motor abilities such as balance, coordination, and strength [24] with activities that demand greater cognitive and coordinative engagement. This could be an effective strategy for structuring AB programs, likely due to the stimulation of executive functions [25]. Moreover, these findings are consistent with those reported by Greco et al. (2024) [26], who demonstrated that structured PA interventions implemented during school hours lead to significant improvements in speed, coordination, and power.

By assessing both completion time and error rate, the RIT test offers insight into cognitive processes such as inhibitory control, attentional regulation, and processing speed, which are involved in top-down cognitive control [27]. In the cognitive tests, all the EGs showed improvement from pre- to post-test across all measures, particularly in terms of faster task completion times, likely due to enhanced cerebral perfusion induced by repeated high-intensity PA over time [28], as well as the stimulation of creative tasks that engage top-down motor planning and organizational functions [29]. These factors may have jointly contributed to the observed outcomes. In addition, the COM group showed improvements compared to the CON group in global cognitive efficiency, evaluated with ITC, which is probably due to a combination of physical and cognitive stimuli provided by this AB protocol [24,26]. Significant reductions in reaction times, processing speed, attention, inhibition, and problem-solving speed, following AB activity, have been found in several other studies [30,31,32].

Regarding the math tests, a significant improvement in arithmetic operation, problem-solving, and total scores were observed across the EGs. This is consistent with Howie et al. (2015) [33], who reported that, when examining acute effects, participants achieved higher math scores after both 10 min and 20 min aerobic AB sessions compared to the control group. In contrast, Mavilidi et al. (2021) [34] reported no significant changes in math performance for the AB group after 8 weeks, with effects comparable to the control group. These differences might indicate that the dose or intensity, which were not evaluated, of ABs in that study were possibly insufficient to elicit measurable improvements in math performance.

The three experimental protocols—fitness-based, creativity-based, and combined—were intentionally designed with distinct exercise characteristics to identify which approach would be most suitable for a student population generally less engaged and motivated during traditional curricular PA.

Although no statistically significant differences were found between the EGs, the study confirms the importance—both physically and cognitively—of increasing school time dedicated to physical activity, regardless of the type of intervention, as the EGs outperformed the CON group in every test [35]. It may be advisable to adopt activities that are sufficiently intense while simultaneously promoting cognitive engagement, such as problem-solving and creativity, during physical execution [7].

This study has several limitations: the total duration of the AB intervention added only one hour to the children’s weekly PA; the teachers’ involvement and enjoyment during the activities was not investigated; children’s extra curricula activities were not investigated during the experimental procedures; and the sample size is not too large to generalize the results.

## 5. Conclusions

The study confirms the effectiveness of integrating ABs within the school context: even a modest weekly increase in PA, when optimally distributed throughout the school curriculum, can produce positive effects on both physical and cognitive domains in students.

The combined approach (COM), which integrates both structured and creative exercises, appears to be the most effective protocol, demonstrating significant improvements in both motor and cognitive skills. This suggests that a mixed-method strategy may represent an optimal model for educational programs, as it concurrently supports physical and cognitive development.

The observed cognitive benefits—such as enhanced attention, reduced response times, and improved problem-solving abilities—are further reinforced by gains in motor performance, underscoring the strong link between physical activity and cognitive function in children.

## Figures and Tables

**Table 1:** Sample characteristics. Data are shown as mean and standard deviation (SD).

	CRE	FIT	COM	CON
**N**	20	20	20	20
**Male**	8	10	11	7
**Female**	12	10	9	13
**Age**	7.97 ± 0.18	7.93 ± 0.26	7.97 ± 0.18	7.42 ± 0.49
**Weight**	31.38 ± 6.75	27.19 ± 3.81	31.77 ± 8.19	31.02 ± 6.48
**Height**	1.30 ± 0.04	1.27 ± 0.05	1.30 ± 0.06	1.28 ± 0.07
**BMI**	20.01 ± 3.59	16.74 ± 1.76	19.38 ± 4.24	18.31 ± 2.64

**Table 2:** Results of MotorFit performance tests. Data are shown as mean and standard deviation (SD).

	FHF-1 (Score)		FHF-2 (Score)		LG (Score)		CB (Score)		TBH (Score)		HB (Score)		VEL (s)		LJ (m)	
	Pre	Post	Pre	Post	Pre	Post	Pre	Post	Pre	Post	Pre	Post	Pre	Post	Pre	Post
**FIT**	3.0 (0.8)	3.8 (0.5) *	3.1 (0.7)	3.9 (0.3) *	3.3 (0.5)	3.3 (0.9)	2.9 (0.8)	3.5 (0.8)	2.4 (0.5)	3.0 (0.6)	3.0 (0.7)	3.4 (0.7)	27.8 (2.9)	24.4 (3.3) *	98.6 (23.5)	117.2 (23.5) *
**CRE**	3.1 (0.7)	3.6 (0.6) *	3.3 (0.8)	3.8 (0.6) *	3.2 (0.6)	3.6 (0.7)	3.2 (0.8)	3.2 (0.8)	2.5 (0.7)	2.7 (0.5)	2.7 (0.8)	3.4 (0.7) *	26.3 (2.8)	25.5 (4.1)	97.9 (16.7)	104.5 (17.8)
**COM**	3.3 (0.9)	3.7 (0.6) *	3.2 (0.9)	3.8 (0.4) *	3.2 (0.6)	3.0 (0.9)	2.8 (0.8)	3.3 (0.8)	2.6 (0.5)	2.7 (0.7)	2.7 (0.6)	3.4 (0.6) *	27.2 (2.5)	25.2 (3.5) *	108.7 (21.9)	122.1 (20.8) *
**CON**	2.9 (0.7)	**2.4 (0.7) ^§^**	3.0 (0.8)	**2.5 (1.0) ^§^**	3.1 (0.6)	3.3 (0.9)	2.8 (0.7)	2.7 (0.8)	2.5 (0.5)	1.8 (0.9)	2.6 (0.6)	**1.9 (0.6) ^§^**	27.2 (2.7)	26.2 (2.8)	103.1 (14.9)	103.7 (13.8)
**EFFECT**																
**Time** **F_(1,76)_**	F = 11.613 *p* = 0.001 * η^2^_p_= 0.133		F = 10.830 *p* = 0.002 * η^2^_p_ = 0.125		F = 0.441 *p* = 0.509 η^2^_p_ = 0.006		F = 3.470 *p* = 0.066 η^2^_p_ = 0.044		F = 0.765 *p* = 0.385 η^2^_p_ = 0.010		F = 6.832 *p* = 0.011 * η^2^_p_ = 0.082		F = 25.971 *p* < 0.001 * η^2^_p_ = 0.255		F = 17.189 *p* < 0.001 * η^2^_p_ = 0.184	
**Interaction** **F_(3,76)_**	F = 8.360 *p* < 0.001 * η^2^_p_ = 0.248		F = 8.690 *p* < 0.001 * η^2^_p_ = 0.255		F = 1.324 *p* = 0.273 η^2^_p_ = 0.050		F = 2.071 *p* = 0.111 η^2^_p_ = 0.076		F = 7.301 *p* < 0.001 * η^2^_p_ = 0.224		F = 10.032 *p* < 0.001 * η^2^_p_ = 0.284		F = 2.738 *p* = 0.049 * η^2^_p_ = 0.098		F = 2.749 *p* = 0.049 η^2^_p_ = 0.098	
**Intervention group** **F_(3,76)_**	F = 8.665 ***p* < 0.001 ^#^** η^2^_p_ = 0.255		F = 9.691 ***p* < 0.001 ^#^** η^2^_p_ = 0.277		F = 1.121 *p* = 0.364 η^2^_p_ = 0.042		F = 2.266 *p* = 0.088 η^2^_p_ = 0.082		F = 5.842 ***p* = 0.001 ^#^** η^2^_p_ = 0.187		F = 14.645 ***p* < 0.001 ^#^** η^2^_p_ = 0.366		F = 0.305 *p* = 0.822 η^2^_p_ = 0.012		F = 2.961 ***p* = 0.037 ^#^** η^2^_p_ = 0.105	
**POST-HOC**																
**FIT vs. CRE**	*p* = 1.000		*p* = 1.000		*p* = 1.000		*p* = 1.000		*p* = 1.000		*p* = 1.000		*p* = 1.000		*p* = 1.000	
**FIT vs. COM**	*p* = 1.000		*p* = 1.000		*p* = 1.000		*p* = 1.000		*p* = 1.000		*p* = 1.000		*p* = 1.000		*p* = 0.895	
**FIT vs. CON**	***p* = 0.001 ^#^**		***p*< 0.001 ^#^**		*p* = 1.000		*p* = 0.162		***p*= 0.003 ^#^**		***p* < 0.001 ^#^**		*p* = 1.000		*p* = 1.000	
**CRE vs. COM**	*p* = 1.000		*p* = 1.000		*p* = 0.527		*p* = 1.000		*p* = 1.000		*p* = 1.000		*p* = 1.000		***p* = 0.044 ^#^**	
**CRE vs. CON**	***p* = 0.002 ^#^**		***p* < 0.001 ^#^**		*p* = 1.000		*p* = 0.162		***p* = 0.013 ^#^**		***p*< 0.001 ^#^**		*p* = 1.000		*p* = 1.000	
**COM vs. CON**	***p* < 0.001 ^#^**		***p* < 0.001 ^#^**		*p* = 1.000		*p* = 0.693		***p* = 0.008 ^#^**		***p* < 0.001 ^#^**		*p* = 1.000		*p* = 0.135	

* = Significant improvement over time (pre- vs. post-intervention); ^§^ = significant worsening over time; **^#^** = significant difference between groups. s = seconds; m = meters.

**Table 3:** Results of cognitive performance. Data are shown as mean and standard deviation (SD).

	ITC (Score)		E OI PO (Score)		T OI PO (s)		E CI PO (Score)		T CI PO (s)	
	Pre	Post	Pre	Post	Pre	Post	Pre	Post	Pre	Post
**FIT**	92.9 (5.2)	97.6 (5.2) *	1.1 (1.1)	0.2 (0.4) *	78.8 (18.3)	69.6 (16.4) *	2.5 (1.8)	0.7 (1.0) *	226.2 (46.2)	204.8 (54.6) *
**CRE**	91.7 (5.1)	98.6 (3.4) *	1.2 (1.4)	0.2 (0.4) *	77.9 (20.1)	64.9 (13.4) *	2.1 (1.8)	0.8 (1.2) *	224.3 (28.7)	183.2 (40.0) *
**COM**	93.2 (4.5)	100.1 (4.0) *	1.0 (0.7)	0.05 (0.2) *	78.0 (14.7)	65.9 (13.4) *	2.3 (1.2)	0.5 (1.2) *	209.9 (51.4)	181.4 (45.9) *
**CON**	92.6 (5.5)	92.5 (5.5)	1.0 (0.9)	0.8 (1.0)	78.1 (20.8)	77.5 (20.8)	2.3 (1.7)	2.0 (1.5)	216.4 (46.0)	222.1 (48.7)
**EFFECT**										
**Time** **F_(1,76)_**	F = 112.310 *p* < 0.001 * η^2^_p_ = 0.596		F = 39.125 *p* < 0.001 * η^2^_p_ = 0.340		F = 35.430 *p* < 0.001 * η^2^_p_ = 0.318		F = 43.884 *p* < 0.001 * η^2^_p_ = 0.366		F = 22.605 *p* < 0.001 * η^2^_p_ = 0.229	
**Interaction** **F_(3,76)_**	F = 14.721 *p* < 0.001 * η^2^_p_ = 0.368		F = 2.842 *p* = 0.043 * η^2^_p_ = 0.101		F = 3.709 *p* = 0.015 * η^2^_p_ = 0.128		F = 3.275 *p* = 0.026 * η^2^_p_ = 0.114		F = 4.859 *p* = 0.004 * η^2^_p_ = 0.161	
**Intervention group** **F_(3,76)_**	F = 2.865 ***p* = 0.042 ^#^** η^2^_p_ = 0.102		F = 1.069 *p* = 0.367 η^2^_p_ = 0.040		F = 0.644 *p* = 0.589 η^2^_p_ = 0.025		F = 1.947 *p* = 0.129 η^2^_p_ = 0.071		F = 1.387 *p* = 0.253 η^2^_p_ = 0.052	
**POST-HOC**										
**FIT vs. CRE**	*p* = 1.000		*p* = 1.000		*p* = 1.000		*p* = 1.000		*p* = 1.000	
**FIT vs. COM**	*p* = 1.000		*p* = 1.000		*p* = 1.000		*p* = 1.000		*p* = 0.789	
**FIT vs. CON**	*p* = 0.356		*p* = 1.000		*p* = 1.000		*p* = 0.692		*p* = 1.000	
**CRE vs. COM**	*p* = 1.000		*p* = 1.000		*p* = 1.000		*p* = 1.000		*p* = 1.000	
**CRE vs. CON**	*p* = 0.430		*p* = 1.000		*p* = 1.000		*p* = 0.289		*p* = 1.000	
**COM vs. CON**	***p* = 0.032 ^#^**		*p* = 0.481		*p* = 1.000		*p* = 0.210		*p* = 0.441	

* = Significant improvement over time (pre- vs. post-intervention); **^#^** = significant difference between groups. s = seconds.

**Table 4:** Results of mathematical test performance. Data are shown as mean and standard deviation (SD).

	OPS (Score)		NJ (Score)		TU (Score)		OT (Score)		MP (Score)		TS (Score)	
	Pre	Post	Pre	Post	Pre	Post	Pre	Post	Pre	Post	Pre	Post
**FIT**	3.3 (1.9)	4.5 (1.6) *	5.5 (1.2)	5.9 (0.2)	5.3 (1.2)	5.6 (0.7)	8.6 (2.7)	9.2 (1.4)	4.0 (1.5)	5.2 (1.3) *	26.9 (6.1)	30.3 (2.9) *
**CRE**	3.0 (1.4)	4.6 (1.3) *	5.8 (4.5)	5.9 (0.2)	5.3 (1.7)	5.6 (0.6)	8.8 (1.8)	9.1 (1.3)	3.9 (1.2)	5.2 (1.1) *	26.9 (3.6)	30.6 (2.4) *
**COM**	3.3 (1.9)	5.2 (0.8) *	5.6 (1.0)	5.9 (0.2)	5.1 (1.5)	5.3 (1.5)	8.7 (2.0)	9.3 (1.2)	3.9 (1.5)	5.8 (0.9) *	26.7 (5.5)	31.4 (3.1) *
**CON**	3.0 (1.3)	3.1 (1.4)	5.2 (1.4)	5.0 (2.0)	4.4 (1.9)	4.7 (2.0)	8.7 (2.0)	8.7 (1.8)	3.8 (1.4)	3.8 (1.2)	25.3 (4.1)	25.1 (5.1)
**EFFECT**												
**Time** **F_(1,76)_**	F = 43.912 *p* < 0.001 * η^2^_p_ = 0.366		F = 1.021 *p* = 0.316 η^2^_p_ = 0.013		F = 2.807 *p* = 0.098 η^2^_p_ = 0.036		F = 2.981 *p* = 0.088 η^2^_p_ = 0.038		F = 75.970 *p* < 0.001 * η^2^_p_ = 0.500		F = 43.610 *p* < 0.001 * η^2^_p_ = 0.365	
**Interaction** **F_(3,76)_**	F = 5.312 *p* = 0.002 * η^2^_p_ = 0.173		F = 0.794 *p* = 0.501 η^2^_p_ = 0.030		F = 0.023 *p* = 0.995 η^2^_p_ = 0.001		F = 0.488 *p* = 0.692 η^2^_p_ = 0.019		F = 11.171 *p* < 0.001 * η^2^_p_ = 0.306		F = 6.151 *p* = 0.001 * η^2^_p_ = 0.195	
**Intervention group** **F_(3,76)_**	F = 3.256 ***p* = 0.026 ^#^** η^2^_p_ = 0.114		F = 3.407 ***p* = 0.022 ^#^** η^2^_p_ = 0.119		F = 2.114 *p* = 0.105 η^2^_p_ = 0.077		F = 0.154 *p* = 0.927 η^2^_p_ = 0.006		F = 3.088 ***p* = 0.032 ^#^** η^2^_p_ = 0.117		F = 4.387 ***p* = 0.007 ^#^** η^2^_p_ = 0.148	
**POST-HOC**												
**FIT vs. CRE**	*p* = 1.000		*p* = 1.000		*p* = 1.000		*p* = 1.000		*p* = 1.000		*p* = 1.000	
**FIT vs. COM**	*p* = 1.000		*p* = 1.000		*p* = 1.000		*p* = 1.000		*p* = 1.000		*p* = 1.000	
**FIT vs. CON**	*p* = 0.208		*p* = 0.142		*p* = 0.195		*p* = 1.000		*p* = 0.191		***p* = 0.042 ^#^**	
**CRE vs. COM**	*p* = 1.000		*p* = 1.000		*p* = 1.000		*p* = 1.000		*p* = 1.000		*p* = 1.000	
**CRE vs CON**	*p* = 0.423		*p* = 0.087		*p* = 0.195		*p* = 1.000		*p* = 0.225		***p* = 0.030 ^#^**	
**COM vs. CON**	***p* = 0.020 ^#^**		***p* = 0.030 ^#^**		*p* = 0.720		*p* = 1.000		***p* = 0.032 ^#^**		***p* = 0.013 ^#^**	

* = Significant improvement over time (pre- vs. post-intervention); **^#^** = significant difference between groups.

## Data Availability

Data will be made available on request.

## Abbreviations

The following abbreviations are used in this manuscript:

MVPA	Moderate-to-vigorous physical activity
PE	Physical Education
PA	Physical activity
ABs	Active Breaks
EGs	Experimental groups
CON	Control group
CRE	Creativity-based AB
FIT	Fitness-based AB
COM	Combined AB
AC-MT 6-11	Test for the Assessment of Calculation and Problem-Solving Skills
RIT	Reynolds Interference Task test
FHF-1	Forward hopping on one foot
LG	Lateral gallop
FHF-2	Alternating forward hopping on one foot
TBH	Throwing a ball with one hand
CB	Catching a thrown ball
HB	Hitting a ball with a tennis racket
VEL	10 × 5 m shuttle run
LJ	Long jump from a standing start
OPS	Operations
NJ	Numerical Judgment
TU	Tens and Units Task
OT	Ordering Tasks
MP	Mathematical Problems
TOTAL SCORE	Final score
OI	Object interference
CI	Color interference
ITC	Index Total Correct
E OI PO	Number of errors in object interference task
T OI PO	Completion time of object interference task
E CI PO	Number of errors in color interference task
T CI PO	Completion time of color interference task
RPE	Rating of Perceived Exertion
